# Modeling an Optimal 3D Skin-on-Chip within Microfluidic Devices for Pharmacological Studies

**DOI:** 10.3390/pharmaceutics14071417

**Published:** 2022-07-06

**Authors:** Estibaliz Fernandez-Carro, Maricke Angenent, Tamara Gracia-Cazaña, Yolanda Gilaberte, Clara Alcaine, Jesús Ciriza

**Affiliations:** 1 Tissue Microenvironment (TME) Lab, Aragón Institute of Engineering Research (I3A), University of Zaragoza, 50018 Zaragoza, Spain; 808895@unizar.es (E.F.-C.); m.l.angenent@student.tudelft.nl (M.A.); 2 Department of Dermatology, University Hospital Miguel Servet, IIS Aragón, 50018 Zaragoza, Spain; tgraciac@salud.aragon.es (T.G.-C.); ygilaberte@gmail.com (Y.G.); 3 Biomedical Research Networking Center in Bioengineering, Biomaterials, and Nanomedicine, CIBER-BBN, Campus Rio Ebro, 50018 Zaragoza, Spain; 4 Institute for Health Research Aragón (IIS Aragón), 50009 Zaragoza, Spain

**Keywords:** skin-on-chip, microfluidic devices, ECM, microbiome, immune system, TEER, dermatology, pharmacological test, toxicological test, cosmetic test

## Abstract

Preclinical research remains hampered by an inadequate representation of human tissue environments which results in inaccurate predictions of a drug candidate’s effects and target’s suitability. While human 2D and 3D cell cultures and organoids have been extensively improved to mimic the precise structure and function of human tissues, major challenges persist since only few of these models adequately represent the complexity of human tissues. The development of skin-on-chip technology has allowed the transition from static 3D cultures to dynamic 3D cultures resembling human physiology. The integration of vasculature, immune system, or the resident microbiome in the next generation of SoC, with continuous detection of changes in metabolism, would potentially overcome the current limitations, providing reliable and robust results and mimicking the complex human skin. This review aims to provide an overview of the biological skin constituents and mechanical requirements that should be incorporated in a human skin-on-chip, permitting pharmacological, toxicological, and cosmetic tests closer to reality.

## 1. Introduction

The skin is the largest organ of the body and plays a key role in various bodily processes. The different essential functions of the skin can either be passive or active. Passive skin functions are related to its barrier function and include the prevention of water loss and the regulation of a relatively constant gas gradient [1,2]. In addition, the skin plays an active role in thermoregulation by means of sweat glands and hair to maintain a constant body temperature, facilitate tactile sensing through embedded pressure sensors, or form an indispensable barrier against external agents. The complex barrier function of the skin is key for maintaining internal homeostasis and protecting the body from harm by external physical, chemical, and biological agents, such as mechanical stresses, ultraviolet radiation, and microorganisms [3]. The human skin is one of the most dynamic organs of the body with a constant skin regeneration ensuring the replacement of the outermost cells that are exposed to the environment. This regeneration is generated by upward moving inner cells derived from the deeper skin layers [4]. The composition of the skin is structurally complex, including a multitude of different cell types to execute the different functions, depending on the exact bodily location. Human skin comprises three distinct structural layers: the epidermis which houses keratinocytes and forms the first line of defense against external agents, the dermis (connective tissue) which provides the skin with most of its mechanical properties and consists mainly of a matrix embedding fibroblasts, and the hypodermis (subcutaneous tissue) responsible for insulation. Understanding the native state and full complexity of the skin, both in a healthy and disease context, is relevant to study pathological and physiological skin conditions. To this end, accurate and physiological representative skin models that mimic the native skin properties and allow for direct drug toxicology studies, disease studies, and better understanding of skin physiology under stressors are required. Though, in recent years, large advancements in the field of skin tissue engineering have been made, in vitro 3D skin models still have several challenges to reproduce the native skin properties and complete function, as yet not fully understood [1,5]. To elucidate the complete physiology of the skin, including its mechanics, signaling pathways, and skin barrier properties, more advanced in vitro models must be developed, not only capturing the structural integrity of the skin layers, but also including other components, such as the immune system, microbiome, or relevant skin appendages. Moreover, innovative sensing technology that allows real-time monitoring will ensure a robust and reliable platform. The use of an optimized in vitro model will pave the way towards novel toxicological analysis of chemical compounds, will aid advanced personalized study of fibrosis, and skin tumors and will allow for the design and investigation of other skin-related pathologies, such as dermatological inflammatory pathology [1].

## 2. Conventional Skin Models Do Not Completely Reproduce Natural Skin

Historically, animal models have been used extensively for the purpose of understanding the skin barrier function and its reaction to topically applied chemical substances [6]. Although animal models have greatly contributed to our basic understanding of skin mechanics, animal experimentation currently presents significant ethical problems with the entry into force in 2013 of the EU Cosmetics Regulation 1223/2009 prohibiting animal testing of cosmetic products [6,7,8], with current research roadmaps asking for reduction, refinement, and replacement of animal experiments, also referred to as 3R research [9]. Moreover, there is limited translatability of animal experimental results due to the inherent differences between human and animal skin physiology [10,11] that poorly predict human skin responses [1]. Thus, half of the drugs approved after animal model studies have shown toxicity to humans [2,12], or higher permeability than in human skin [13,14]. Consequently, there is a need for the development of alternative skin models that better represent human native skin, also reducing the use of experimental animals.

Human ex vivo skin explants are an alternative to animal testing. Skin biopsies from healthy or unhealthy samples can be maintained in culture, therefore providing the full complexity of the tissue in vivo. However, human ex vivo skin explants are hampered by donor variability, availability, and biological limitations [1,15]. Consequently, recent research has focused on developing human skin equivalents (HSEs) that either recapitulate the full thickness skin or separate layers of the skin in two-dimensional (2D) or three-dimensional (3D) culture systems [16]. Primary or immortalized human cells in 2D cultures are usually grown as a monolayer in a controlled flat environment with a single cell type or multiple cell types, sometimes involving cell patterning [17]. Although 2D culture conditions are relatively simple systems, easy to establish, and allow high-throughput screenings [16,18,19], their application is limited due to their lack of native tissue complexity and the tendency of cells to flatten and stretch. This morphology abnormality causes cellular alterations such as proliferation, differentiation, apoptosis, or altered gene expression [18,19], therefore not mimicking the in vivo cellular skin microenvironment conditions. The 3D cell cultures more closely reproduce human skin physiology, more accurately recapitulating mechanical and chemical signals as well as morphology [18], and overcoming 2D cell culture limitations. The 3D skin constructs, often developed with natural extracellular matrix molecules or synthetic polymers as scaffold, allow cell–cell and cell–matrix interactions. Alginate, collagen, chitosan, fibrin, hyaluronic acid, elastin, poly(ethylene glycol), polycaprolactone, poly(vinyl alcohol), or polylactic acid are common materials used in scaffolds, forming a hydrogel that simulates the dermis with keratinocytes seeded on top, mimicking the epidermis [4]. Collagen is frequently used since it is the most abundant component of the extracellular matrix (ECM). However, the static conditions of these 3D in vitro models do not satisfactorily mimic the natural skin dynamics, even when they can accurately capture the permeability properties of human skin better than under static conditions [19]. Moreover, scaffolds show rapid degradation and excessive hydrogel contraction, providing a shortened lifetime and preventing their applicability over time [4]. Another drawback is the lack of immune system, such as dendritic cells interspersed in the dermis and epidermis with important implications in skin pathologies [20,21], or the absence of blood vessels, preventing the dynamic transport of nutrients and growth factors, waste removal, or cell migration. In fact, the incorporation of vascularization prolongs the skin model lifespan [4,20,22].

Currently, there are various commercial human skin models that only include the epidermal skin layer, such as SkinEthic^TM^ (EpiSkin, L’Oréal Lyon France), EST1000V^®^ (CellSystems, Troisdorf, Germany), Open Source Reconstructed Epidermis (OS-Rep) (Henkel, Düsseldorf, Germany), StratiCELL (StratiCELL, Les Isnes, Belgium), StrataTestV^®^ (Stratatech, Madison, WI, USA), or more recently the LabCyte Epi-model (LabCyte, Gamagori, Japan), and other models simulating both dermis and epidermis, such as Vitrolife-Skin^TM^ (Kyoto, Japan), Phenion^®^ (Henkel, Düsseldorf, Germany), EpiDerm-FT^TM^ (Mattek, Ashland, OR, USA), CELLnTEC (CELLnTEC, Berne, Switzerland), and Biomimiq (Biomimiq, Leiden, The Netherlands) skin models (Table 1) [4,6]. However, challenges remain, and an ideal model incorporating all the skin components with physiological accuracy has yet to be constructed. The reported tissue engineered skin models still show reduced barrier properties compared to in vivo human skin and often lack immune cells and other microenvironmental skin constituents that normally reside inside and outside the tissue [23]. As an answer to solve issues related to current 3D skin model approaches, the combined approach of tissue engineering and use of microfluidics represents an alternative. In fact, recently, an increasing number of microfluidic platforms that try to solve these issues, called skin-on-chip (SoC) devices, are being investigated.

## 3. Biological Requirements for Skin-on-Chip (SoC) Devices

**Figure 1:**
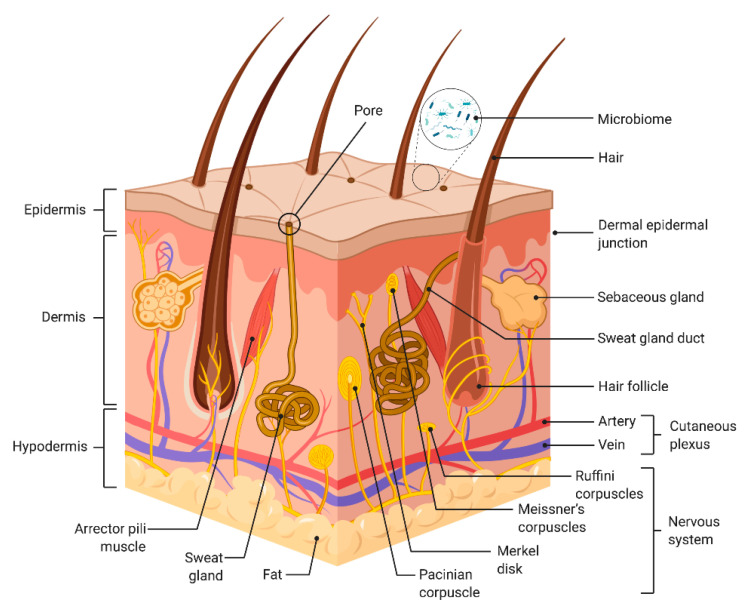
Schematic representation of the different skin layers. Adapted from ‘Anatomy of the skin’, by BioRender.com (2022). Retrieved from https://app.biorender.com/biorender-templates, accessed on 9 June 2022.

Cells grown within microfluidic chips, commonly referred to as ’organ-on-chip’ (OoC), are dynamic models, where the microenvironment is controllable [24]. Skin-on-chip (SoC) technologies can control parameters related to topography, fluidic *shear stress*, and culture perfusion [6]. SoC devices are made with a porous substrate that separates the microchannels and the wells where the tissue is deposited. This design allows the study of specific tissue barrier functions and tissue–tissue interactions [4]. Structurally, the skin contains different cell types whose function is synchronized to maintain its functionality and integrity, forming three distinct layers: the epidermis, the dermis, and hypodermis (Figure 1) [25]. Each layer has distinct structural properties, cellular organization, and function, varying the thickness of each compartment depending on the body location. Thus, the thickest skin body regions, such as the hand palms and foot soles, are hairless, with an additional epidermal layer called the ‘stratum lucidum’, while skin covering the upper back contains the thickest dermal layer, lacking *stratum lucidum* [26].

**Figure 2:**
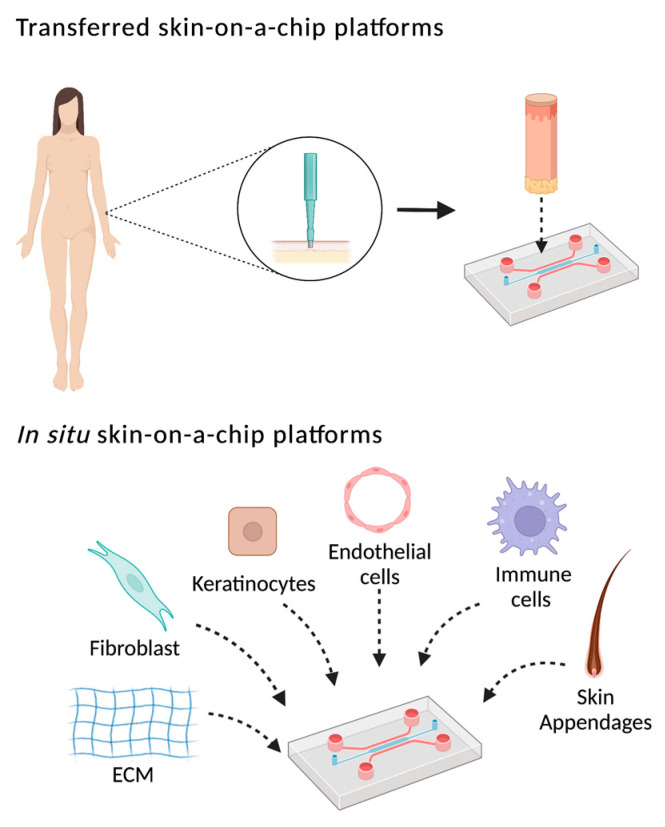
Schematic representation of SoC approaches. Created with BioRender.com.

According to Risueño et al., SoC can be classified into two different approaches: those where tissues from a biopsy or HSE is inserted directly into the device, or those where tissue is generated in situ on the chip (Figure 2) [4]. However, this classification does not discern between the type of material and manufacture of the device, the tissue composition with dermis, epidermis, vascular irrigation, or immune system, the maintenance of the tissue, or the mechanical requirements. Therefore, an analysis of the different components required in an ideal SoC model should be considered (Table 2).

### 3.1. The Epidermis

Epidermis, the first line of defense against external agents and the interface to exchange substances with the exterior [25], is formed by keratinocytes, melanocytes, immune system cells, and the microbiome on the surface. This skin layer is avascular and includes five distinct layers: the *stratum basale* (the deepest portion of the epidermis), *stratum spinosum*, *stratum granulosum*, *stratum lucidum* (only present in some areas of the body), and *stratum corneum* (the most superficial portion of the epidermis). The complete migration from *stratum basale* to *stratum corneum* last at least 14 days, and an additional 14 days are required for the complete transit through the cornified layer to the outermost epidermis [27,28], therefore establishing the time required to form an epidermis 3D model. Melanocytes forming melanin units associated with keratinocytes keep a melanocyte/keratinocyte ratio of 1/10 [29], a factor also considered when developing epidermal 3D models.

One of the first 2D models generated with epidermal keratinocytes grew confluent keratinocytes in a feeder-layer of fibroblasts and the addition of epidermal growth factor (EGF) [30,31]. However, due to the presence of serum in the medium and difficulty to distinguish various cellular growth stages, the model was mainly limited to rapid production of large numbers of keratinocytes and studies of cellular growth. Subsequently, another serum-free epidermal skin model emerged [32], but relied again on molecular controllers of epidermal differentiation. Thus, autocrine culture conditions without growth factors emerged as a new alternative, based on the keratinocytes’ endogenous production [33]. However, an air–liquid interface to expose the outermost tissue layers to air, necessary in the development of granular and cornified skin layers, was not implemented. Air stimulates differentiation and induces epidermal stratification and barrier formation [34], implying that the medium must be supplied to cells from the bottom of the tissue construct. In fact, the comparison of transcriptomes shows similar morphology and gene expression between 3D keratinocyte multilayered models grown in the air–liquid interface and human skin, but not with 2D keratinocyte culture where differentiation markers and apoptotic genes are suppressed [35]. However, 3D skin models still express a lower number of transcription factors, cell surface receptors, and secreted proteins compared to human skin [35,36]. These differences can be attributed to the absence of dermis that influences cells from epidermis and vice versa, altering transcriptomics and the lack of cell–cell interaction with other cell types such as immune system cells in epidermis that can also alter cellular pathways. Current 3D organotypic systems are still based in human-derived keratinocytes seeded on an inert surface area and grown with the air–liquid interface, allowing reproducibility and stability of the model over long periods of time, such as SkinEthic^TM^ [37]. However, they lack Langerhans cells in the spinous and granulomatous layer, or the dermal layer, precluding cell–cell dermis–epidermis interactions, or the skin microbiome on the epidermis surface, whose imbalance can lead to various diseases, and therefore compromise the results in these models.

Recent epidermis-on-chip devices have achieved advances mimicking basal, spinous, granular, and cornified layers with an architecture close to human skin with similar expression of keratin-10, keratin-14, involucrin, filaggrin, or loricrin. They efficiently integrate transepithelial electrical resistance (TEER) determination, non-invasively monitoring integrity and differentiation of the keratinocyte monolayer. Moreover, the system can completely distinguish between irritant and non-irritant compounds, meeting the OECD demand of sensitivity 80%, specificity 70%, and accuracy 75% [38]. However, although irritation tests noteworthily show significant cell death, decreases in paracellular permeability, increases in inflammatory cytokines, such as IL-6 and TNF-alpha, significant damage to epidermal morphology, effects on tight junctions, or monolayer barrier disruption corroborated by TEER, the lack of other cells in epidermis or dermis does not accurately represent the human skin permeability and irritation. For example, they lack myeloid cells from the immune system that are activated in the presence of irritants, triggering an immediate response by releasing pro-inflammatory mediators that cause inflammation [39].

### 3.2. The Dermal–Epidermal Junction

The dermal–epidermal junction (DEJ), the interface between epidermis and dermis holding both layers together, keeps structural skin integrity and prevents injury from external shear forces [5]. The DEJ provides a unique microenvironment characterized by rete ridges required for the epidermal stem cell niches [5], with a porous structure that allows selective exchange of fluids and cells [27], playing a central role in establishing cell polarity and determining the growth direction of epidermis. The dynamic interface between epidermal ridges and dermal papillae ensures an augmented shear resistance and increased surface area that allows increased paracrine diffusion between the skin layers, underlining the importance of including an accurate DEJ in skin models to achieve a complete mature skin construct [5,40]. DEJ structure and signaling processes are governed by a broad network of intracellular, extracellular, and transmembrane proteins, such as keratins and hemidesmosomes [5,41]. Structurally, keratinocytes of the epidermal basal layer are anchored to collagen type IV, collagen type XVII, laminins, and integrins which make up a specialized ECM, the lamina densa. In turn, the lamina densa is secured to the dermis through an interaction with fibrils composed of collagen type VII. The reconstruction of this complex assembly of proteins is crucial for achieving epidermal integrity [42].

Current bioengineered skin models generally include a flat interface design as the DEJ, rather than the corrugated DEJ structure found in native skin. Since the DEJ is hypothesized to play a significant role in establishing the cellular microenvironments of stem cell niches in the skin, embedding a spatially similar structure in skin models is bound to increase the physiological accuracy of in vitro skin structures. Dynamic microfluidic environments with continuous medium perfusion were shown to have a positive effect on the establishment of a functional DEJ with enhanced deposition of collagen types IV, VII, and XVII. In fact, a SoC model with a dynamic perfusion and ventilation system resulted in a pluristratified skin construct including a mature DEJ [42], therefore confirming that engineering a DEJ to achieve an optimized topology that mimics native rete ridges may further improve the physiological accuracy and viability of in vitro skin constructs [43]. To date, engineering strategies employed to create an effective DEJ include photolithography, laser structuring, electrospinning, 3D printing, or a combination [5,43]. Adequate representation of the DEJ represents an interesting approach for testing anti-aging treatments in cosmetic applications, since the fibers anchoring dermis to epidermis degrade, reducing dermal papillae density and leading to a 20–35% DEJ flattening [5].

### 3.3. The Dermis

The dermis accounts for the bulk of the skin and it is characterized by its pliability, elasticity, and tensile strength mechanical protection, thermal regulation, and regulation of fluid homeostasis [27]. Dermis is arranged in two connective tissue layers: the upper papillary and deeper reticular layer, differing in their tissue organization, cell density, and nerve and vascular patterning [28]. From the sub-papillary plexus, the boundary between the thin papillary layer molding contours of epidermis composed of highly vascular loose connective tissue, and the deeper thick reticular dermis with dense connective tissue, capillaries extend into the dermal papillae in a loop-wise manner, thereby supplying the epidermis by subsequent diffusion [44]. Thus, dermis is mainly composed of collagen (primarily type I and type III) and elastic fibers (elaunin horizontally and oxytalan perpendicularly arranged) that provide the mechanical properties of the tissue. Besides the structural components, the dermis contains fibroblasts, providing the structural skin ECM, macrophages, and mast cells [28].

**Figure 3:**
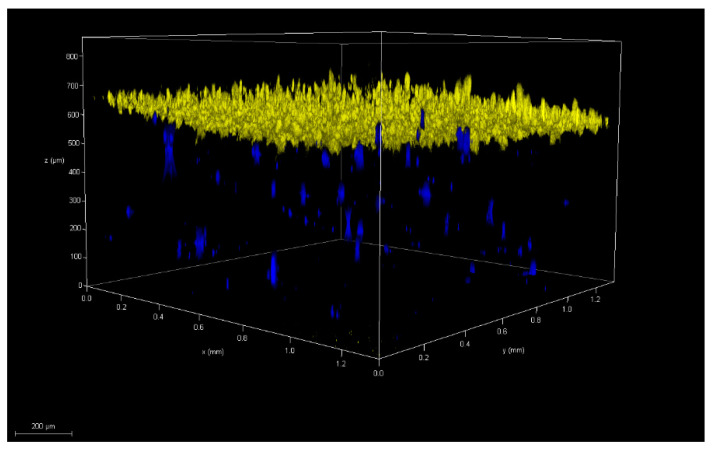
Skin model recapitulating full skin thickness with labeled epidermal layer formed by HaCat cells on top (yellow) and labeled dermal layer formed by primary dermal fibroblast (blue) below within a microfluidic device.

Skin models that recapitulate the full skin thickness must include a dermal layer (Figure 3). Various approaches to build up an artificial layer of connective tissue in vitro have been reported, where collagen hydrogels are the gold standard [45]. For example, SoC models with dermis, simulated with primary human fibroblasts embedded in collagen, and epidermis with human keratinocytes on top of the collagen hydrogel, separated by a membrane within a PDMS device showed that the optimal type of collagen for a skin-on-chip model is rat tail collagen [46]. Adding gravity flow to the device, hydrogel shrinkage was reduced, due to the different evaporation ratio, liquid supplementation from surrounding media, and the diffusion plus convection transport from the medium to the hydrogel, also enhancing keratinocyte differentiation [47]. This system represents a breakthrough in SoC design, being suitable for drug testing with drugs such as *Curcuma longa* leaf extract, a natural anti-aging skin cosmetic that at 50 μg/mL concentration in this system achieves skin recovery after 7 days of treatment [48]. However, the model still lacks endothelial cells forming the vascular system, not reproducing the cellular barrier to reach the dermis.

The generation of the dermis in microdevices is not easy to achieve, since the behavior of hydrogels in small-dimension devices hinders the obtention of a controlled gel surface. Alternatives such as the injection of the hydrogel through a channel over a porous membrane separating the lower channel, then generating a flat surface with an upper parallel flow controlled by syringe pumps to seed keratinocytes, is a breakthrough to optimize a standardized and reproducible dermis system [49]. The possibility to add endothelial cells recovering the medium channel in the future opens the way for further promising experiments. Other approaches have generated microfluidic devices able to stretch the skin by adding a magnet component that allows deformation of the device, creating uniaxial tension in the tissue. This model consisting of fibroblasts embedded in a type I collagen hydrogel with keratinocytes on the hydrogel surface allows skin aging studies [50]. However, a flat surface does not accurately represent a DEJ which could cause a different behavior under stretch. In fact, the lack of immune cells and skin microbiota in the aforementioned models precludes close mimicking of the human skin, since the immune system can trigger different reactions with unknown substances, while drugs can alter microbiota, leading to diseases such as inflammatory dermatoses or acne, among others. Moreover, under stretch stress, blood vessels are also stretched, maybe leading to an extravasation of immune cells in the blood through the endothelial layer. The addition of a TEER system to those devices could also help to non-invasively monitor the integrity of the epidermis in real time.

### 3.4. The Skin Vasculature

Skin microcirculation regulates skin homeostasis, thermoregulation, and blood pressure, directs inflammatory responses, and deliver nutrients and other systemic factors [51], allowing for longer tissue survival and maintenance [52]. Thermoregulation by means of vasodilation increases skin blood flow and facilitates heat dissipation, whereas vasoconstriction decreases the heat loss from the body [27]. The main components of dermal blood vessels are microvascular endothelial cells, that synthesize and secrete chemokines and cytokines, activating the immune system in the migration of leukocytes to sites of inflammation and participating in ECM formation [53]. Therefore, it is important to incorporate vascular structures in in vitro SoC models. Thus, induction of inflammation and edema with different doses of TNF-α into the dermis of a PDMS device with a porous PET membrane and endothelial layer at the lower dermis shows damage to cell tight junctions caused by inflammation, affecting the endothelial cell cytoskeleton by creating intercellular gaps that increase skin permeability. The treatment with dexamethasone reduces the increased permeability induced by TNF-α, reflecting the clinical, pharmacological, or cosmetic applications of this approach [54]. However, the absence of a DEJ boundary means it does not completely represent human skin.

Some improvements in reproducing the vascular system have been achieved by using cell-coating and accumulation techniques [55]. Blood vessel-like cultures in 3D printed bioreactors have been controlled with perfused medium through a fibronectin and gelatin nano-film dermal compartment, modulating vascular endothelial growth factor (VEGF), hypoxia-inducible factor 1A (HIF1A), and matrix metalloproteinases/tissue inhibitors of metalloproteinases (MMPs/TIMPs) gene expression. The modulation of MMPs/TIMPs prevented ECM degradation, avoiding the appearance of empty gaps where uncontrolled angiogenesis could occur, with induction of vessel formation through HIF1A and VEGF expression. With this flow system, skin thickness was increased, therefore increasing filaggrin expression and TEER values [52], more closely reproducing the DEJ. These remarkable advances must be considered in future skin-on-chip devices. Nevertheless, the SoC models lack an immune system such as Langerhans cells, macrophages, or immune cells present in the vascular system, precluding the study of cell migration through the skin layers when damage occurs, or the microbiome, that can affect permeability and other mechanical requirements, such as stretching and compression force to reach a fully advanced model.

### 3.5. Immune System

The skin protects against infections by pathogens, bacteria, fungi, and viruses, by the physical barrier of the epidermis and the innate and adaptive immune system, including skin-residing immune cells and biomolecules [39]. Immune cells include myeloid cells, such as Langerhans cells, macrophages, dermal dendritic cells, or eosinophils, responsible for immediate response to inflammatory conditions by producing pro-inflammatory mediators, and lymphoid cells, such as T-lymphocytes, B-lymphocytes, and natural killer cells [39]. Achieving a skin-on-chip model that can correctly simulate the immune system will help to understand their cellular mechanisms and to simulate skin diseases and study possible treatments.

Thus, ultraviolet stimulation of a PDMS SoC device with dermis and epidermis separated by a porous membrane and perfused by gravity, containing leukocytes, showed the presence of the adhesion molecule CD31 in the vascular endothelial layer and a reduced permeability, with increased doxorubicin toxicity, especially in keratinocytes [20]. Irritation studies produced an inflammatory reaction that caused cytokine release by immune cells, reduced after anti-inflammatory treatment. The release of cytokines at the site of inflammation activated endothelial cells leading to the recruitment of leukocytes present in the microchannel flow [53]. Other SoC models with macrophages cocultured with fibroblasts, endothelial cells, and keratinocytes seeded on Matrigel within a SoC were able to simulate wounds by TNF-α addition, and a further increase in IL-6 and IL-8, altering cell–cell junctions of linear vascular structures and increasing abnormal vascular formation capacity. The presence of M2 macrophages enhanced IL-8 but after administration of dexamethasone, cytokine levels decreased, restoring normal vascular organization [56]. However, the interaction of other immune cells and skin cells remains unclarified. Other SoC models with whole untreated blood from patients and skin extracted from patient microbiopsies have determined the immune system response against *S. aureus*, analyzing the inflammation generated by neutrophils [57], since *Staphylococcus aureus* and *Streptococcus pyogenes* are the most common causes of skin and soft tissue infections. However, how patient sample handling after removal affects tissue cells and disrupts their physiological functions, the variability between patients’ samples with significant amounts of neutrophils, or the role of skin intrinsic immune cell populations must be clarified.

Immune system integration also allows the study of other dermatological diseases, such as atopic dermatitis (AD). AD is caused by epithelial barrier disruption and dysregulation of the immune Th2 response, with increased IL-4 and IL-13 that trigger the activation of JAK1/JAK2/TYK2-STAT6 and -STAT3 pathways, inhibiting the expression of filaggrin, loricrin, and involucrin. Thus, induced atopic dermatitis by IL-4 and IL-13 in a full thickness SoC showed greater disruption of epidermis, decreasing the expression of barrier proteins [58], particularly affecting the stratum corneum, with large intracellular spaces. However, the role of the cutaneous microbiota should not be overlooked since the overabundance of *S. aureus* and *S. epidermis* bacteria is related to AD [59].

### 3.6. Microbiome

Human skin is home to a vast number of bacteria, fungi, and viruses, giving rise to the skin microbiota. Skin microorganisms have essential functions such as protection against invading pathogens, education of the immune system, and breakdown of natural products [60], being continuously exposed to the surrounding environment, which influences the diversity of existing bacterial cells [61]. The microbiome is beneficial to the body and serves as physical barrier to prevent invasion by pathogens [61], with antimicrobial peptides (AMPs), mostly released by epidermal keratinocytes and controlled by the skin microbiota that disrupt bacterial membranes [39,61]. In fact, different microbiome compositions have been studied according to the location of the skin, with different skin pathologies related to alterations in the microbiome. However, the microbiome is often forgotten in SoC models with a lack of reports regarding integrated microbiota. The integration of microbiota in SoC devices would allow the study of personalized treatments for pathologies caused by a dysfunction of the microbiome, by growing within the SoC model the specific microbiome population from the affected patient [60].

Resident microorganisms present on the skin are not considered pathogenic in undamaged skin, but opportunistic infections may occasionally occur due to idiopathic causes. Thus, the human skin commensal *Staphylococcus epidermidis*, *Cutibacterium acnes*, and *Malassezia furfur*, and the transient pathogenic *Staphylococcus aureus*, grown over keratinocytes seeded on fibroblast-embedded fibrin matrix dermis in a Transwell^®^ insert, show that oil exceeding physiological levels leads to development of *C. acnes* or *M. furfur*, responsible for inflammatory acne or seborrheic dermatitis and pityriasis versicolor, respectively [62]. Moreover, the imbalance of *C. acnes* and peroxidized squalene treatment in a 3D model with a single epidermal layer is able to reproduce acne-prone skin, with an increase in inflammatory factors, decrease in claudin-1, and reduced epidermis integrity [63,64]. Although these models are a good initial approach to study cutaneous microbiome and pathologies, the addition of other microorganisms from the skin and sebaceous glands, flow, or other mechanical requirements for the model would more closely represent human skin, allowing permeability studies or simulating different pathologies caused by alterations in the microbiota.

### 3.7. Nerves

Skin includes both somatic sensory and sympathetic autonomic nerve fibers. The sensory fibers function as receptors of touch, pain, temperature, itch, and mechanical stimuli, comprising specialized receptors such as the Merkel disks, Pacinian, Meissner’s, and Ruffini corpuscles [28]. The sympathetic nerve fibers control the tone of the vasculature, pilomotor stimulation of the hair root, and apocrine gland secretion, innervating vascular smooth muscle, sweat glands, the arrector pili muscle of hair follicles, and the sebaceous glands [28]. After culturing skin explants with primary sensory neurons, tissue innervation is observed after 10 days, both in the dermal and epidermal layers. This leads to an increase in epidermal thickness, cell density, and explant quality, resulting in optimal skin homeostasis, showing the influence of the nervous system in skin models [65]. Several other skin models have focused on the regeneration and growth of neurons and innervation of the tissue. Thus, neuritis development from the dermal to the epidermal layer in an in vitro human skin equivalent model exhibited sensitivity to topical application of the compound capsaicin, recognized by keratinocyte receptors, transmitting the signal along the neurites, and propagating a calcium wave [66]. Another approach combining liver iPS and nerve cells in different channels embedded in collagen, with primary keratinocytes in a third channel, showed that high doses of tretinoin, a cosmetic used for exfoliating treatments, caused loss of epidermal thickness and loss of involucrin expression in stratum corneum, while capsaicin administered topically produced a dose-dependent calcium release increase in nerve cells. Furthermore, lactic acid, used in anti-wrinkle cosmetics, administered simultaneously with strontium chloride suppressed neuronal activation. Interestingly, indicators of hepatotoxicity were detected after topical administration of hepatotoxic compounds at lower levels than in liver cell monolayer cultures, demonstrating the role of the skin barrier [67]. However, the lack of sensitivity studies in some models, together with the variability of patient samples, limits the model. Microfluidic devices with engineered skin could at least also provide immune system or mechanical requirements, reducing economic cost and more closely mimicking the natural skin.

**Table 2:** Human SoCs reported with different biological requirements.

Biological Requirement	Device Material	Cell Types	Main Characteristics	Refs.
Epidermis	4PMMA layers	NHK	1 µm pore size PET membrane (density of 2 × 10^6^ pores/cm^2^). Differentiation of different layers of the epidermis. Correctly distributed epidermal proteins. Efficient debubble chip. Integration of perfusion and TEER.	[38]
Epidermis and dermis	2 PDMS layers	HK HDF	Transwell^®^-cut membrane. Differentiation of different layers of the epidermis. Gravity flow helps to reduce shrinkage of the hydrogel.	[46,47]
Epidermis and dermis	PDMS	Human primary keratinocytes Human primary fibroblasts	Devices fabricated by soft lithography. Useful for analyzing the effects of drugs or cosmetic products.	[48]
Epidermis and dermis	8 vinyl layers, PDMS layer 2PMMA layers	HaCaT HDF	PC membrane (5 µm pore size). Epidermis and dermis layer. Fibrin hydrogels with a thickness like that of human dermis, fairly homogeneous. Automatization and standardization of the hydrogel loading process. Presence of *shear stress*.	[49]
Epidermis and dermis	2 PDMS layers	HEK HDF	Permanent magnet inserted into a cavity. Presence of mechanical forces.	[50]
Epidermis, dermis, and vascular layer	2 PDMS layers separated by membrane	HaCaT HDF HUVEC HL-60	Presence of immune system. Perfusion (10 μL/min) or gravity-driven flow.	[53]
Epidermis, dermis, and vascular layer	PDMS channels	HaCaT HS27 HUVEC	Fabricated using soft lithography. PET membranes (obtained from Transwell^®^). Model for the study of inflammation and edema.	[54]
Dermis, vascular layer, and immune system	PDMS	HDF HUVECs M1 and M2 macrophages	Devices fabricated by soft lithography. Three main channels: 2 laterals for 2D monolayer and inner channel for 3D coculture. Simple model to simulate early inflammation phase. Useful for analyzing the effects of drugs	[56]
Epidermis and dermis	PDMS	Human volunteer’s abdominoplasty	Devices fabricated by soft lithography. Human full thickness skin sample. Blood loading channel 1.5 mm (1 mm inlet/outlet channels). Crossing the endothelial membrane simulated by the filter system. Useful device for migration studies.	[57]
Epidermis and dermis	2 PDMS layers	NHEK HDF	0.4 μm porous membrane. Prevention of shrinkage of the dermal scaffold by functionalization of the surface.	[58]
Epidermis, nerves, and liver	PDMS	HEK hNSC hiPSC-HEP	Devices fabricated by soft lithography. Four sections for each cell type. Representation of the effect of substances at different levels of the organism. Reproducibility.	[67]

### 3.8. Skin Appendages

The skin contains hair, nails, eccrine and apocrine sweat glands, and sebaceous glands participating in protection, sensory reception, thermoregulation, or lubrication, and should also be considered in a SoC model. In fact, complete hair follicle (HF) units grown ex vivo on a chip maintaining the perifollicular epidermis, dermis, and sebaceous glands preserve the basement membrane, connective tissue, and the dermal papilla of the follicle, with the hair shaft enlarged [68]. However, HF structure is affected, decreasing the number of nuclei in the central and proximal HF. Although dynamic culture demonstrates the possibility of tissue life elongation, HF units perpendicular to skin layers should be integrated to obtain a complete full thickness, avoiding the immersion of the visible part of the hair to represent the native skin more closely. This approach would allow cell–cell interaction between different cell populations, resulting in a more advanced and significant model. Recently, hair has been developed from human pluripotent stem cells (hPSCs) within a complex skin with layered epidermis, fat-rich dermis, pigmented HF with sebaceous glands, and a network of sensory neurons and Schwann cells. PSC differentiation is directed towards epidermal precursors enveloped by dermal precursors, obtaining, in approximately 70 days, an HF organoid spaced across the epidermis with similar mammalian morphology to normal skin. Although the organoid reveals the presence of chin, cheek, or outer ear skin, no resident skin immune cells are detected. In addition, organoids show pigmentation, hyaline cartilage, a layer of lipid-rich adipocytes surrounding the culture, and a basic nervous system like that of an 18-week human fetus [69]. This is the most advanced model currently described, but models where adult hair of the scalp is adequately represented in dynamic conditions has not been reported yet, even when the demand for hair treatments with the aim of preventing hair loss is increasing.

## 4. Mechanical Components

In addition to biological factors, mechanical factors that help to mimic the natural skin microenvironment should be considered [70]. Mechanical forces such as compression, tension, or shear affect cell differentiation, proliferation, phenotype, migration, and apoptosis, being perceived by cells as mechanical stimuli, with final mechanotransduction into biological responses [71,72]. Therefore, mechanical features such as flow and mechanical *shear stress* should be integrated in a SoC. Moreover, other components such as integration of sensors for real-time monitoring should be considered (Table 3).

### 4.1. Flow and Shear Stress

Blood vessels, an essential element for an advanced in vitro SoC, not only act as a flow conduit, but also enhance graft durability, being essential in angiogenic studies, angiostatic drug studies, and cancer research [73,74,75,76]. Furthermore, an adequate vasculature is a step towards the implementation of hair follicles, sweat glands, nerves, or immune system [77]. However, SoCs with adequate microvasculature are scarce and existing models have limited stability and durability ranging from 7–14 days, since skin vessel formation can be hindered by dermal contraction caused by fibroblasts, ECM proteolysis, MMP and TIMP signaling cascades from keratinocytes, and the presence of other cell types, avoiding the optimal environment where endothelial cells proliferate, migrate, form branching tubes of microvasculature, and survive over time [52]. In fact, the perfusion by external flow of microvasculatures developed from seeded stem cells or endothelial cells generates inaccessible spontaneous and random vessel formation, hindering nutrient transport and limiting the accuracy of vascularized models [77].

**Figure 4:**
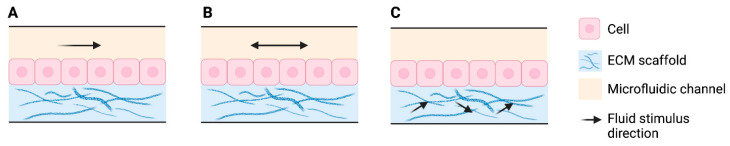
Flow inducing laminar (**A**), pulsatile (**B**), or interstitial (**C**) *shear stress*. Created with BioRender.com.

The inclusion of dynamic flow in SoCs partly replaces the role of blood vessels, by continuously supplying nutrients and removing waste products and metabolites from the system, extending lifespan [42]. Thus, dynamic flow can support the maintenance of vascularized SoCs over a long period of time, improving epidermal morphogenesis, differentiation, and an enhanced skin barrier function, with a more mature basement membrane [42,52]. Upon the introduction of flow within microfluidic systems, *shear stress* is induced, generated by laminar, pulsatile, or interstitial flow either in the cell surface or in the cells attached to the ECM (Figure 4). *Shear stress* provides mechanical stimuli to the cells with subsequent effects on cell adhesion, mechanics, morphology, and growth, relevant in SoC models [78]. In fact, although keratinocytes are not exposed to fluid in the postnatal period, during embryogenesis skin is exposed to amniotic fluid, with different multidirectional transport mechanisms relevant at the early growth phases [79]. Moreover, the behavioral pattern of human epidermal keratinocytes exposed to *shear stress* is different to cells cultured in static models, improving cell viability by *shear stress* [80]. However, the flow-induced mechanostimulus has not yet been extensively studied in skin.

**Figure 5:**
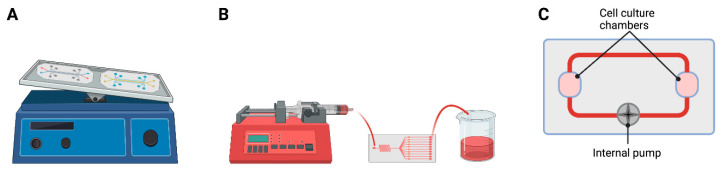
Methods and equipment to generate *shear stress*. Tilt movement in the rocker (**A**), external syringe pump (**B**), and internal pump within the chip (**C**). Created with BioRender.com.

*Shear stress* can be generated by passive delivery with a rocker, external pumping with a syringe pump or peristaltic pump, or internal pumping integrated within the chip (Figure 5) [78,81]. External pumping can be programmed with precise and accurate flow rates, exposing cells to laminar *shear stress* at specific flow rates. *Shear stress* is calculated with the following equation [78,79]:

$$Shear\;stress,\;T=\frac{6\mu Q}{h^{2}w}$$

where *Q* represents flow rate, *µ* fluid viscosity dependent on the medium, *h* channel height, and *w* channel width. Thus, flow rates from 0.025–0.4 μL/min vary the proliferation rate and cell viability of human epidermal keratinocytes. However, *shear stress* also induces mechanoresponses in keratinocytes, generating damage to the cytoskeleton at high *shear stress*, and inducing reorganization at low *shear stress*, conferring mechanoresistance and mechanotransduction with enhanced levels of E-cadherin and ZO1, indicative of an increased cell adhesion [79,80]. *Shear stress* also affects cell migration in wound models, providing faster healing under an optimal *shear stress* due to the migration of fibroblasts [82]. However, how other cell types behave under *shear stress* in SoC models has not yet been determined.

### 4.2. Compression and Stretch

Compression or stretching studies are scarce, with aging being the main subject of study since a microsized surface simulates the first stages of wrinkles. Some studies show that a microfluidic device with induced 10% uniaxial stretch by using a magnet can deform the device and the tissue, generating wrinkles after 7 days and exerting mechanotransduction that reduces collagen, fibronectin, and keratin-10 production [50]. Computational models explain how uniaxially compressive stress generates those wrinkles, determined by the geometry and material properties of the skin [83]. Stretching tension in vascularized SoCs [77] promotes the stratification and differentiation of the epidermis, with thicker epidermis similar to human skin, enhancing the basement membrane and increasing collagen concentration and cell density in the dermis [84]. The transcription factor YAP, the predominant modulator of epithelial proliferation, is translocated from the nucleus to the cytoplasm when mechanical force is exerted. However, proliferation only occurs in short-term mechanical stretching. In long-term mechanical stretching, translocation of YAP occurs with an H3K27me3 increase and expression of its methyltransferase EZH2. This epigenetic event arrests epithelial proliferation, being reversible if a methyltransferase inhibitor compound is administered, restoring proliferation [85]. In vivo, higher equibiaxial cyclic stretch induces an increase in vascular permeability, increased potassium efflux, and, in turn, sodium influx. The changes in sodium and potassium concentrations cause endothelial cell contraction by weakening the endothelial barrier, allowing inflammatory factors to penetrate areas of injury, and leading to increased inflammation with increased ECM fibroblast secretion and the development of hypertrophic or keloid scars. To prevent keloid scarring, β-hydroxybutyrate has been proposed as a therapeutic candidate, inhibiting the stretch-induced Ca^2+^ response, the opening of the K_ATP_ channel, and the subsequent activation of the NLRP3 inflammasome (activated by intracellular K^+^ release) [86]. Reproducing these events in SoC models with the different actors involved would shed light on these skin biological processes.

### 4.3. Integrated Sensors

The integration of sensors into SoC models allows continuous monitoring of cell behavior and their response to administered drugs [87]. Traditionally, skin structure has been assessed by invasive histological staining, but microfluidic systems easily allow a continuous, non-invasive, real-time monitoring by integrating biosensors [4]. Thus, integrated biosensors within OoC devices can monitor cell microenvironment, such as temperature, pH, humidity, oxygen, and cell behavior, such as proliferation, viability, cell adhesion, or metabolic activity, quantifying hydrogen peroxide, glucose, lactate, or other cell release [88]. With current advances, miniaturized integrated sensors can be fully integrated in the device and are used on-chip, monitoring change in the sample directly, or off-chip, analyzing the fluid passing through the channels [82]. However, integrated sensors in SoC devices have focused mainly on transepithelial electrical resistance (TEER), without development of other biosensors.

Epithelial monolayers’ integrity can be non-invasively quantified in real time by TEER readouts, embedding electrodes directly into the chip [83]. TEER quantification is based on either ohmic resistance of the tissue or impedance over a spectrum of frequencies [84]. Keratinocyte differentiation in air–liquid culture increases electrical resistance over time due to formation of tight junctions in stratum corneum, dropping after detergent treatment. Thus, for example, an automated TEER system (IMOLA-IVD) implemented in a SoC model mimicking epidermis can show how SDS causes loss of epithelial barrier integrity, lacking dermis or mechanical stimulus, such as tension [89]. Commercial electrodes used in TEER are often chopstick type and present a lack of reproducibility due to manual handling without uniform current density in large tissue sizes [90]. Modular architecture and custom-made integrated tetrapolar electrodes can overcome these issues, showing a loss of epithelial integrity by 0.2% SDS in SoC with fibroblasts embedded in a commercial scaffold, covered by primary keratinocytes [91].

**Table 3:** Human SoCs reported with different mechanical requirements.

Mechanical Requirement	Device Material	Cell Types	Main Characteristics	Ref
Perfusion TEER	PMMA 1 µm pore size PET membrane	NHK	Differentiation of different layers of the epidermis. Correctly distributed epidermal proteins. Efficient debubble chip.	[38]
Pumpless, gravity driven	PDMS Transwell^®^-cut membrane	Primary human keratinocyes HDF embedded in rat tail collagen hydrogel	Differentiation of different epidermal layers. Epidermal and dermal layers. Gravity flow helps to reduce shrinkage of the hydrogel. Cell–cell and cell–matrix interactions.	[46,47]
Perfusion	PDMS	Human keratinocyes Human fibroblasts	Presence of mechanical forces and their effect on cell behavior. Epidermal and dermal layers.	[50]
Parallel flow controlled with syringe pump	PDMS PMMA Vinyl layer, PC membrane	HaCaT HDF embedded in fibrin hydrogel	Epidermal and dermal layers. Hydrogels with a thickness like that of human dermis. Automatization and standardization of the hydrogel loading process. Complementation between mathematical model and experimental model.	[49]
Gravity driven	PDMS PET membranes	HaCaT HS27 fibroblasts HUVECs	Epidermal, dermal, and vascular layers. Study of inflammation and edema.	[54]
Perfusion gravity driven	PDMS	HaCaT or primary keratinocytes HDF HUVECs HL-60	Epidermal, dermal, and vascular layer.Immune system presence.	[53]
Gravity driven	PDMS porous membrane	NHEK HDF	Epidermal and dermal layers. Prevention of shrinkage of dermal scaffold by functionalization of the surface.	[58]
Pulsatile flow, micropump	PDMS	EpiDerm^®^, human skin explant, and hair follicle explant	Epidermis, dermis, and skin appendage (hair follicle). Two simultaneous microfluidic circuits.	[68]
Perfusion TEER	PMMA Porous membrane	N/TERT-1 HDF	Epidermal and dermal layers. Real-time monitoring.	[42]
Syringe pump	PDMS	NHEK	Epidermal layer.	[80]
Syringe pump	PDMS	HaCaT	Epidermal layer. Study of mechanotransduction.	[79]
Double-side perfused	PDMS, polystyrene, and membrane or scaffold	HDF HEKn	Epidermal and dermal layers. Uniform current density. Real-time assessment. Low cost.	[91]

The integration of temperature sensors into SoC models has aided in the evaluation of drug penetration through the skin. Thus, after taping with leucoplast, the diffusion of a hydrophilic drug, such as caffeine, in a vaseline-based cream formulation into native and sensitized skin has shown that as skin becomes more sensitive, its permeability is increased, providing information close to traditional in vivo and ex vivo animal models [92]. Electrochemical sensors for the detection of O_2_ consumption and lactate production have also been reported but in breast cancer spheroid models, with platinum sensors integrated in the microfluidic device [93]. An O_2_ sensor is based on the reduction of dissolved molecular oxygen in an electrolyte, while a lactate sensor is based on an enzymatic reaction with lactate oxidase. A similar device could be adapted for SoC. However, the combination of several sensors integrated into one single device would be more interesting, with physical sensors for the measurement of pH and O_2_ and temperature sensors and electrochemical immunobiosensors for the detection of biomarkers, also allowing observation under microscopy with continuous and automated real-time monitoring. For example, a multiorgan human heart-and-liver organ-on-chip was able to test the hepatotoxic drug acetaminophen (APAP) and the chemotherapy drug doxorubicin (DOX), in addition to hyperthermia treatments. Electrochemical immunobiosensors were impedance-based functionalized with antibodies for the detection of released proteins, while pH and O_2_ sensors and temperature sensors consisted of physical sensors and sondes, respectively. After administration of different doses of APAP and DOX causing toxicity in hepatocytes and cardiomyocytes, respectively, albumin decreases, or glutathione S-transferase α (GSK-α) and creatine kinase MB (CK-MB) increases, could be quantified by immunobiosensors. In addition, hyperthermia treatments induced a cell metabolism decrease attributed to cell necrosis/apoptosis, monitored by pH, O_2_, and temperature sensors. A similar design in SoC would allow monitoring non-invasive physical and biochemical parameters of the cellular microenvironment [87]. However, this type of design is very complex.

## 5. Conclusions

Dermatology is the area in which advanced 3D in vitro models have been most developed and widely used with applications that range from toxicity testing to drug discovery in human disease models. The prohibition to perform animal testing for the development of new products for cosmetic applications had a huge impact in the development of novel in vitro skin models. For that reason, scientific and industrial communities in dermatology are the most prepared for new advances of in vitro models. Therefore, current in vitro skin models are already well accepted in the industry for toxicity testing towards a new technological solution. However, they lack the integration of complex functions of human skin provided by the incorporation of the immune system and microbiome, among others. Building on existing full thickness skin models, innovative sensing technology and microfluidic devices would allow for ensuring a robust, reliable, economical, and scalable platform. Reported SoC models still fail to represent complete native skin and need further research to make progress.

**Figure 6:**
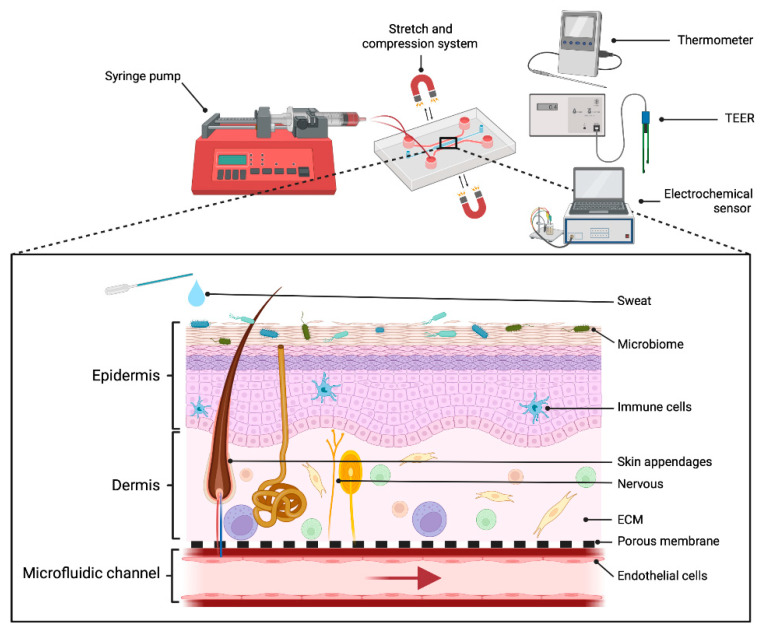
Biological and mechanical requirements of an optimized SoC. Adapted from “Anatomy of the skin”, by BioRender.com (2022). Retrieved from https://app.biorender.com/biorender-templates, accessed on 27 June 2022.

Shrinkage of hydrogel representing dermis due to fibroblast contraction is one of the major bottlenecks in SoC development, with no complete representation of dermis composition. New hydrogels with composition closer to ECM represent one of the main challenges to mimic cell behavior of natural human skin, such as metabolic activity, migration patterns, proliferation viability, and receptor expression. However, SoC models must also face other challenges since a combination of a wide range of biological and mechanical requirements should be implemented (Figure 6). Among biological requirements, resident as well as circulating cells from the immune system, or microorganisms and sweat on the skin surface, should be taken into account, since they can modify drug mechanisms when applied in the model. Current SoC models do not implement those agents, especially the microbiome, even when everyone has a unique and specific population that interacts with the immune system. This interaction regulates pathogen recognition, barrier function, immune response, or the evolution of skin diseases, and would allow the generation of specific skin disease types, such as atopic dermatitis, among many others that are currently unavailable, and the exploration of off-target effects of a drug in the presence of the immune system and microbiome. In addition, common allergic reactions, cancer, inflammatory, or autoimmune skin diseases could be explored without the requirement of animal testing. Moreover, the use of patient-derived elements, such as ECM or immune system cells, paves the way for personalized medicine, allowing study of the pathologies of each individual, taking into account their needs and investigating the most appropriate treatment.

Analysis and characterization of state-of-the-art SoCs is mainly limited to optical and fluorescence microscopy accompanied by the application of cell staining and labeling techniques. The major drawbacks of these methods are that only a single measurement is possible, often requiring the termination of the experiment. Moreover, labels can interact unspecifically with cells and substances under test. Other analytical techniques such as HPLC are not suitable due to the low sample volumes available. Label-free and continuous real-time analysis of cell viability parameters remains one of the most important unresolved technical challenges in advancing SoC models, such as the currently used sensors integrated into OoCs such as electrochemical and optical sensors to monitor oxygen, pH, glucose, and lactate. In addition, transepithelial/transendothelial electrical resistance (TEER) has been instrumental in elucidating cell metabolism and response to external stimuli. However, the application of commercially available cell culture analysis systems remains limited.

Finally, scaling up would facilitate the rapid adoption of the novel technology and aid in the transition from the research and development phase to the commercial phase. Factors to be considered for SoC will be related to the incorporation of automatic, closed loop devices that regulate continuous monitoring and parameter control of the chips, reducing manual work and alleviating chip handling burden for anticipated end-users. If skin-on-chip devices are successfully scaled up to commercial demand, the cost, the required biological resources, and the time related to product testing in pharmaceutical and cosmetic industries will be greatly reduced. Overall, the use of microchips to this end would result in considerable savings in the process of experimental data acquisition.

## Figures and Tables

**Table 1:** Commercial human skin models.

Represented Layers	Commercial Model	Application
Epidermis	SkinEthic^TM^ (EpiSkin, L’Oréal Lyon France)	Skin irritation Skin corrosion Medical devices UV exposure DNA damage Bacterial adhesion Omics Permeability
	Open Source Reconstructed Epidermis (OS-Rep) (Henkel, Düsseldorf, Germany)	Skin irritation Skin corrosion
	StratiCELL (StratiCELL, Les Isnes, Belgium)	Skin aging Barrier function Damage related to light Acute inflammation Pigmentation Pollution
	StrataTestV^®^ (Stratatech, Madison, WI, USA)	Skin irritation Skin corrosion Toxicological assessments
	LabCyte Epi-model (LabCyte, Gamagori, Japan)	Skin irritation Skin corrosion
Epidermis and dermis	Vitrolife-Skin^TM^ (Kyoto, Japan)	Skin irritation Skin corrosion
	Phenion^®^ (Henkel, Düsseldorf, Germany)	Skin physiology Skin biochemistry Clinical dermatology Transdermal drug delivery studies Wound healing Toxicological assessment of chemicals Analysis of environmental effects on skin physiology
	EpiDerm-FT^TM^ (Mattek, Ashland, OR, USA)	Anti-aging Wound healing Skin hydration UV protection
	CELLnTEC (CELLnTEC, Berne, Switzerland)	Skin irritation Skin corrosion Toxicological assessments Omics
	Biomimiq (Biomimiq, Leiden, the Netherlands)	Toxicological assessments Drug development Omics
